# Ferritin and Soluble Transferrin Receptor Beyond Inflammation: Independent Associations with Fasting Glucose and Metabolic Syndrome in a Central Asian Cohort

**DOI:** 10.3390/diagnostics16172773

**Published:** 2026-08-28

**Authors:** Dana Kaldarkhan, Karlygash Sadykova, Ainash Oshibayeva, Gulnaz Nuskabayeva, Gulzira Baimakhanova, Malika Raimova, Nigora Alikulova, Nodira Khamidova, Shoira Isanova, Dinara Azizkhojayeva, Ainur Turmanbayeva, Kanatzhan Kemelbekov

**Affiliations:** 1 Faculty of Medicine, Khoja Akhmet Yassawi International Kazakh-Turkish University, Turkestan 160000, Kazakhstan; dana.kaldarkhan2024@ayu.edu.kz (D.K.); karlygash.sadykova@ayu.edu.kz (K.S.);; 2 Department of Neurology and Traditional Medicine, Tashkent State Medical University, Tashkent 100109, Uzbekistan; 3 Department of Neurology, Samarkand State Medical University, Samarkand 140103, Uzbekistan; 4 Department of Pediatrics-1, South Kazakhstan Medical Academy, Shymkent 160019, Kazakhstan

**Keywords:** metabolic syndrome, ferritin, soluble transferrin receptor, gamma-glutamyl transferase, fasting glucose, biomarkers, Kazakhstan

## Abstract

**Background/Objectives**: Metabolic syndrome (MetS) is a major global public health concern associated with an increased risk of cardiovascular disease and type 2 diabetes mellitus. Iron metabolism has been implicated, but the storage (ferritin) and functional (soluble transferrin receptor, sTfR) axes are rarely assessed within a single model. We aimed to determine whether both axes are independently associated with glycaemia and MetS and to evaluate their diagnostic value. **Materials and Methods**: A cross-sectional study was conducted in 297 adults (72.8% women) from Turkestan, Kazakhstan. Hierarchical regression models with sequential adjustment were applied for age, sex, body mass index (BMI), high-sensitivity C-reactive protein (hs-CRP), dietary inflammatory index (DII), *HFE* H63D genotype, and gamma-glutamyl transferase (GGT). Path analysis and receiver operating characteristic (ROC) analysis were also performed. **Results**: Ferritin and sTfR were independently associated with fasting glucose (β = 0.27 and 0.73, respectively) and metabolic syndrome (OR = 1.52 and 4.53, respectively). When analyzed separately, neither biomarker reached statistical significance for MetS (ferritin: OR = 1.20, *p* = 0.140; sTfR: OR = 1.93, *p* = 0.072). Ferritin demonstrated no discriminatory ability in men (AUC = 0.500, 95% CI 0.370–0.630) and modest discrimination in women (AUC = 0.652, 95% CI 0.571–0.732). GGT accounted for approximately 28% of the ferritin–glucose association in a path-analytic model. Ferritin, GGT, and HbA1c levels increased progressively even among individuals with 0–2 MetS components. **Conclusions**: Both the storage and functional axes of iron metabolism provide independent information on glycaemic status, but their associations become apparent only when assessed simultaneously. Ferritin alone is not suitable as a diagnostic marker for metabolic syndrome.

## 1. Introduction

Metabolic syndrome (MetS), a cluster of abdominal obesity, impaired glucose metabolism, atherogenic dyslipidaemia, and elevated blood pressure, is a major risk factor for type 2 diabetes mellitus (T2DM) and cardiovascular disease (CVD) [1]. Meta-analyses of prospective cohort studies have demonstrated that MetS is associated with increased risks of all-cause mortality and cardiovascular events [2]. The association with T2DM is even stronger: the risk increases proportionally with the number of MetS components and exceeds a tenfold higher risk in individuals with four or more components [3]. However, the current diagnostic criteria have an important limitation: they are binary. Individuals with only one or two MetS components are classified as metabolically healthy, despite carrying an elevated risk of developing T2DM and CVD. Identifying laboratory biomarkers that become abnormal before the diagnostic threshold is reached may therefore improve early risk stratification and overcome this limitation.

Beyond the traditional MetS components, increasing attention has been directed toward biomarkers of iron metabolism, although their role in MetS remains incompletely understood. Meta-analyses of observational studies have shown that elevated serum ferritin is associated with approximately a 1.7-fold higher risk of MetS [4]. Prospective cohort studies have linked higher ferritin concentrations to an increased risk of incident T2DM [5]. Mechanistically, excess free iron possesses pro-oxidant properties and accumulates in the liver and pancreatic β-cells, where it may impair insulin signalling and glucose homeostasis [6,7].

Nevertheless, the clinical utility of ferritin is limited for two main reasons. First, ferritin is an acute-phase reactant and may reflect systemic inflammation rather than iron status per se, particularly in individuals with MetS. Second, ferritin primarily represents iron storage and does not capture the functional availability of iron. In contrast, the soluble transferrin receptor (sTfR) reflects functional iron demand at the cellular level and is relatively unaffected by acute inflammation. sTfR reflects the expression of transferrin receptor 1 (TfR1). TfR1 is expressed on erythroid precursor cells. When hemoglobin synthesis decreases due to iron deficiency, transferrin receptors are shed from the cell surface and released into the circulation, resulting in elevated serum sTfR levels. In clinical practice, serum ferritin is commonly used to assess iron status. However, the advantage of sTfR over ferritin is that it is less affected by chronic inflammatory conditions [8]. However, studies evaluating sTfR have produced inconsistent findings. Some investigations reported no association between sTfR and MetS but observed positive associations with insulin resistance [4]. The EPIC-Potsdam study found no significant association with incident T2DM [5]. Other studies suggested that the relationship between sTfR and T2DM may depend on the presence of obesity [9]. Because ferritin and sTfR reflect complementary aspects of iron metabolism, evaluating both biomarkers simultaneously may help reconcile these discrepancies.

Within this biological framework, the liver occupies a central position as the primary iron storage organ and a key site of insulin resistance. Gamma-glutamyl transferase (GGT), a marker of glutathione turnover, oxidative stress, and hepatic steatosis, has consistently demonstrated dose-dependent associations with both MetS and T2DM [10]. These observations suggest that the relationship between iron metabolism and glycaemic dysregulation may be mediated, at least in part, through a liver-dependent pathway involving oxidative stress.

Most previous studies have been conducted in European and East Asian populations. Meanwhile, Central Asia is experiencing a rapid increase in the prevalence of obesity and MetS [11]. Furthermore, the International Diabetes Federation (IDF) recommends ethnicity-specific waist circumference thresholds, and dietary patterns, quantified using the Energy-adjusted Dietary Inflammatory Index (E-DII), have been independently associated with MetS [12]. However, the combined effects of dietary inflammatory potential and iron metabolism biomarkers have rarely been investigated in the Kazakh population.

Our previous genetic studies further motivated the present investigation. In a systematic review and meta-analysis including 17 studies, we found that the *HFE* p.C282Y polymorphism was not associated with MetS components, arguing against a major role of the classical iron-sensing pathway as an independent determinant of metabolic risk [13]. Subsequently, in a pilot whole-exome sequencing study conducted in the Kazakh population, the strongest genetic associations were observed not in iron-sensing genes but in genes involved in iron transport (*TF*) and hepcidin regulation (*TMPRSS6*). Notably, the association between the *TMPRSS6* haplotype and fasting glucose was not mediated by any conventional iron biomarker—including ferritin, serum iron, transferrin, transferrin saturation, or sTfR—despite a persistent direct genetic effect [14]. These findings suggest that the influence of iron-related genetic variation on glycaemic regulation may operate through mechanisms not captured by standard iron biomarkers, with hepatic function and systemic inflammation representing the most plausible intermediate pathways. The present study was designed to test this hypothesis at the biomarker level by incorporating GGT and hs-CRP directly into the analytical model.

Accordingly, this cross-sectional study had four objectives: (1) to determine whether iron storage (ferritin) and functional iron status (sTfR) are independently associated with fasting glucose and MetS after sequential adjustment for age, sex, body mass index, systemic inflammation (hs-CRP), dietary inflammatory potential (E-DII), the *HFE* H63D genotype, and the hepatic marker GGT; (2) to decompose the association between ferritin and fasting glucose into direct and GGT-mediated pathways; (3) to evaluate the incremental diagnostic value of iron metabolism biomarkers beyond a conventional clinical model; and (4) to explore whether these biomarkers increase already within the subclinical range (0–2 MetS components), before the formal diagnosis of MetS is established. Given the cross-sectional design, all findings should be interpreted as associative rather than causal.

## 2. Materials and Methods

### 2.1. Ethical Approval

The study protocol was approved by the Ethics Committee of Khoja Ahmed Yassawi International Kazakh–Turkish University (Turkestan, Kazakhstan). All participants provided written informed consent before enrolment. The study was conducted in accordance with the principles of the Declaration of Helsinki. Ethical approval was granted on 13 May 2025 (Protocol No. 41).

### 2.2. Study Design and Participants

This analytical cross-sectional study was conducted among adults attending regularly scheduled appointments at the Clinical Diagnostic Center of Khoja Akhmet Yassawi International Kazakh–Turkish University between January and December 2025. Participants were recruited consecutively during routine clinic visits. Eligible participants were aged ≥18 years, provided written informed consent, and had complete data on iron metabolism biomarkers, anthropometric and biochemical measurements, dietary assessment, and *HFE* H63D genotyping. Exclusion criteria were: acute or recent infectious illness within the preceding three months; chronic conditions requiring immediate medical intervention; clinical evidence of anaemia (haemoglobin < 130 g/L in men or <120 g/L in women, ferritin < 15 µg/L, or transferrin saturation < 16%).

Documented hepatitis or liver/gallbladder disease identified by structured medical history interview; and reported alcohol misuse on structured questionnaire. Fewer than 7% of participants reported any alcohol use, consistent with low alcohol prevalence in this Central Asian cohort.

### 2.3. Clinical and Laboratory Assessments

Anthropometric examinations included measurements of height, body weight, waist circumference (WC), and calculation of body mass index (BMI). Blood pressure (BP) was measured using an automated sphygmomanometer after a 5 min resting period. Dietary data were collected using a structured food frequency questionnaire (FFQ) covering major pro-inflammatory and anti-inflammatory food groups. The Energy-adjusted Dietary Inflammatory Index (E-DII) was calculated from FFQ responses according to the method of Shivappa et al. [15], using population-specific mean intakes as the reference.

Venous blood samples were collected in the morning after an overnight fast of at least 8 h, and serum was separated by centrifugation. HbA1c was measured in EDTA-anticoagulated whole blood.

Fasting plasma glucose (mmol/L) was measured using the enzymatic hexokinase/glucose-6-phosphate dehydrogenase method. HbA1c was determined by a turbidimetric inhibition immunoassay and reported as both % (NGSP/DCCT-aligned) and mmol/mol (IFCC). High-density lipoprotein cholesterol (HDL-C, mmol/L) was measured using a homogeneous enzymatic colorimetric assay, and triglycerides (mmol/L) by an enzymatic colorimetric (GPO-PAP) method. Alanine aminotransferase (ALT, U/L) was determined by a kinetic UV assay according to the IFCC reference procedure, and gamma-glutamyl transferase (GGT, U/L) by an enzymatic colorimetric assay (IFCC).

Serum iron (µmol/L) was measured by a FerroZine-based colorimetric assay without deproteinization, and unsaturated iron-binding capacity (UIBC, µmol/L) by the corresponding colorimetric method. Ferritin (µg/L), transferrin (g/L), soluble transferrin receptor (sTfR, mg/L), and high-sensitivity C-reactive protein (hs-CRP, mg/L) were quantified using particle-enhanced immunoturbidimetric assays.

Internal quality control was performed during each analytical run using manufacturer-supplied control materials at two concentration levels.

### 2.4. Genotyping

Genotyping of the *HFE* p.His63Asp (H63D; rs1799945) polymorphism was performed using real-time polymerase chain reaction (real-time PCR) on a QuantStudio™ 5 Real-Time PCR System (Applied Biosystems, Thermo Fisher Scientific, Waltham, MA, USA).

Genotyping was carried out according to the manufacturer’s standard TaqMan^®^ SNP Genotyping Assay protocol (Applied Biosystems, Thermo Fisher Scientific). A no-template control (NTC), containing nuclease-free water instead of genomic DNA, was included in each run. Genomic DNA was diluted to a working concentration of 2 ng/µL, and 5 µL of DNA solution (10 ng DNA per reaction) was added to each reaction, following the manufacturer’s recommendations. Reaction mixtures were prepared according to the standard Thermo Fisher Scientific protocol for TaqMan SNP Genotyping Assays.

Genotypes were assigned using TaqMan™ Genotyper Software 1.7.1 (Applied Biosystems, Thermo Fisher Scientific) based on the fluorescence intensities of the FAM and VIC dyes with automatic sample clustering.

### 2.5. Definition of Metabolic Syndrome

Metabolic syndrome was defined according to the Harmonized Joint Interim Statement [16]. Participants were classified as having MetS if they met at least three of the following five criteria: waist circumference ≥ 94 cm for men or ≥80 cm for women; triglycerides ≥ 1.7 mmol/L or corresponding lipid-lowering therapy; HDL cholesterol < 1.03 mmol/L for men or <1.29 mmol/L for women or corresponding treatment; blood pressure ≥ 130/85 mmHg or antihypertensive treatment; and fasting plasma glucose ≥ 5.6 mmol/L or glucose-lowering therapy. For each participant, the total number of MetS components (0–5) was also recorded.

### 2.6. Statistical Analysis

The primary analysis used hierarchical linear regression with sequential inclusion of covariate blocks: Model 0 (M0), age, sex, and BMI; M1, M0 plus ferritin; M2, M1 plus sTfR; M3, M2 plus log-transformed hs-CRP; M4, M3 plus energy-adjusted Dietary Inflammatory Index (E-DII) and *HFE* H63D genotype; and M5, M4 plus log-transformed GGT. Thus, M0 represented the basic adjustment model, M1 evaluated the storage iron marker alone, and the functional iron marker (sTfR) was introduced from M2 onwards.

Because residuals showed non-normality and heteroscedasticity, robust HC3 standard errors and corresponding 95% confidence intervals (CIs) were calculated. Associations with MetS were examined using logistic regression. Model discrimination was assessed by the area under the receiver operating characteristic curve (AUC), whereas calibration was evaluated using the Hosmer–Lemeshow goodness-of-fit test, the Brier score, and calibration plots.

The incremental value of iron biomarkers was assessed using the likelihood ratio test (improvement in model fit) and DeLong’s test (improvement in discrimination), which address complementary aspects of model performance. The stability of ferritin and sTfR estimates across sequential adjustment models is presented in the Supplementary Materials (Figure S1). Multiple testing for secondary outcomes was controlled using the Benjamini–Hochberg false discovery rate (FDR) procedure, whereas no correction was applied to the predefined primary outcome.

The association between ferritin and fasting glucose was decomposed into direct and indirect (GGT-mediated) effects using path analysis with bootstrap confidence intervals based on 5000 resamples. The *HFE* H63D genotype was analysed under a dominant genetic model (G allele carriers vs. C/C homozygotes), as no G/G homozygotes were identified. An extended iron panel, including TSAT, serum iron, and transferrin, was available for a subset of participants and was analysed as a secondary analysis (Table S1).

*Exploratory subclinical analysis.* A post hoc analysis evaluated whether biomarker concentrations increased progressively across the continuum of metabolic risk. Spearman trend tests were performed for ferritin, GGT, HbA1c, and sTfR according to the number of MetS components, both in the entire cohort and in participants without MetS (0–2 components).

Multiple testing corrections were applied as follows. The primary outcome (fasting glucose) was analysed without correction, as it was the single pre-specified primary endpoint. Secondary continuous outcomes (HDL-C, triglycerides, waist circumference, SBP, and DBP) were corrected using the Benjamini–Hochberg false discovery rate (FDR) procedure separately within each marker (ferritin and sTfR), yielding five tests per marker. The binary MetS outcome was analysed without correction as a single confirmatory binary endpoint. The non-glycaemic MetS sensitivity analysis is treated as exploratory and reported without FDR correction, with explicit labelling of its inferential status.

To examine whether the observed associations were independent of the glycaemic component of MetS, a sensitivity analysis was performed using a non-glycaemic MetS composite outcome, defined as the presence of ≥2 of the following 4 criteria: abdominal obesity (WC ≥ 94 cm in men or ≥80 cm in women), hypertriglyceridaemia (≥1.7 mmol/L), low HDL-C (<1.03 mmol/L in men or <1.29 mmol/L in women), and elevated blood pressure (SBP ≥ 130 mmHg or DBP ≥ 85 mmHg)

Software. Statistical analyses were performed in R version 4.6.1 using the packages dplyr 1.2.1, tidyr 1.3.2, stringr1.6.0, ggplot2 4.0.3, patchwork, sandwich 3.1.1, lmtest 0.9-40, car 3.1-5, broom 1.0.13, performance 0.17.1, pROC1.19.0.1, mediation 4.5.1, and gtsummary 2.5.1.

## 3. Results

### 3.1. Participant Characteristics

A total of 297 participants were included in the study, of whom 120 (40.4%) met the diagnostic criteria for MetS. Women predominated in the cohort (72.8%). Participants with MetS were significantly older than those without MetS (median age 52.5 vs. 44.0 years, *p* < 0.001) and, as expected, exhibited significantly less favourable values for all MetS components (all *p* < 0.001; Table 1). Among iron metabolism markers, serum ferritin concentrations were substantially higher in participants with MetS than in those without MetS (65.9 vs. 31.6 µg/L; *p* < 0.001). GGT (27.0 vs. 18.0 U/L; *p* < 0.001), hs-CRP (2.2 vs. 1.4 mg/L; *p* = 0.002), HbA1c (5.7% vs. 5.4%; *p* < 0.001), and the E-DII score (0.14 vs. 0.07; *p* = 0.002) were also significantly higher in the MetS group. In contrast, HFE H63D carrier frequency did not differ between groups (*p* = 0.540).

The extended iron panel (serum iron, transferrin, and TSAT) was performed in a randomly selected subsample of 207 participants owing to the high cost of reagents; selection was not based on metabolic risk status. Although randomly selected, participants with and without extended panel data differed in several baseline characteristics (age 51 vs. 40 years, BMI 29.3 vs. 27.2 kg/m^2^, fasting glucose 4.94 vs. 4.78 mmol/L, ferritin 53.5 vs. 29.9 µg/L, and MetS prevalence 45.4% vs. 28.9%), suggesting possible chance imbalance (Table S1). Since the subsample was randomly selected, data are more likely missing completely at random (MCAR) rather than by a risk-dependent mechanism, supporting the validity of complete-case analyses within this subsample. Secondary analyses of the extended panel are therefore treated as exploratory.

### 3.2. Correlation Structure

**Figure 1:**
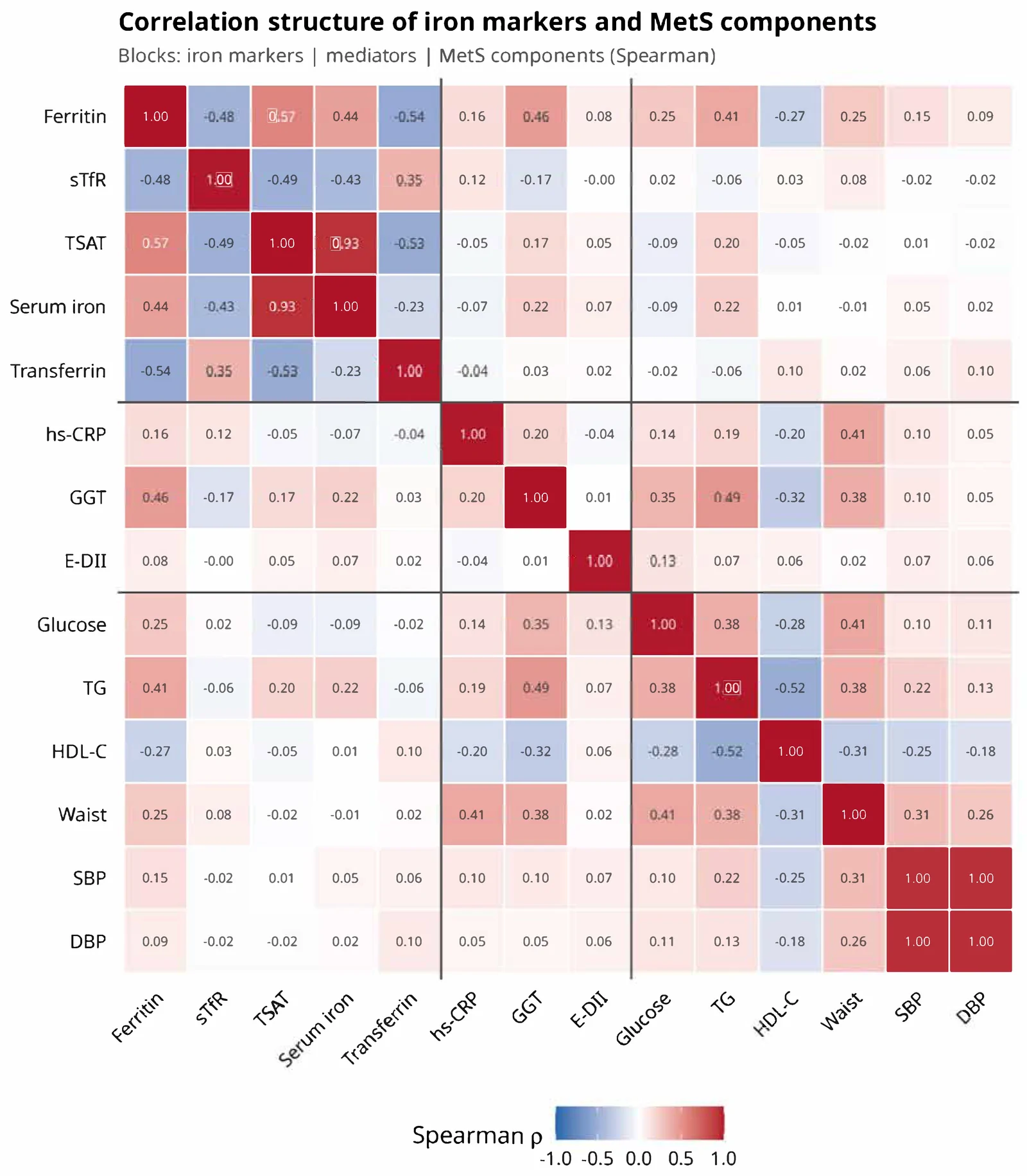
Correlation structure of iron markers and MetS components (Spearman). Blocks: iron markers|mediators|MetS components.

Spearman correlation analysis demonstrated that ferritin, as a marker of iron storage, was positively correlated with transferrin saturation (TSAT; ρ = 0.57) and serum iron (ρ = 0.44) (Figure 1). Ferritin also showed positive correlations with the hepatic-metabolic cluster, including GGT (ρ = 0.46), triglycerides (ρ = 0.41), and fasting glucose (ρ = 0.25).

As expected from iron physiology, ferritin and sTfR were inversely correlated because ferritin reflects body iron stores, whereas sTfR represents functional cellular iron demand.

### 3.3. Both Axes of Iron Metabolism Are Independently Associated with Fasting Glucose

Hierarchical linear regression analysis demonstrated that both ferritin and sTfR remained independently associated with fasting glucose throughout all sequential adjustment models. These associations persisted after adjustment for age, sex, BMI, systemic inflammation, dietary inflammatory potential, *HFE* genotype, and liver function, with robust HC3 standard errors.

**Figure 2:**
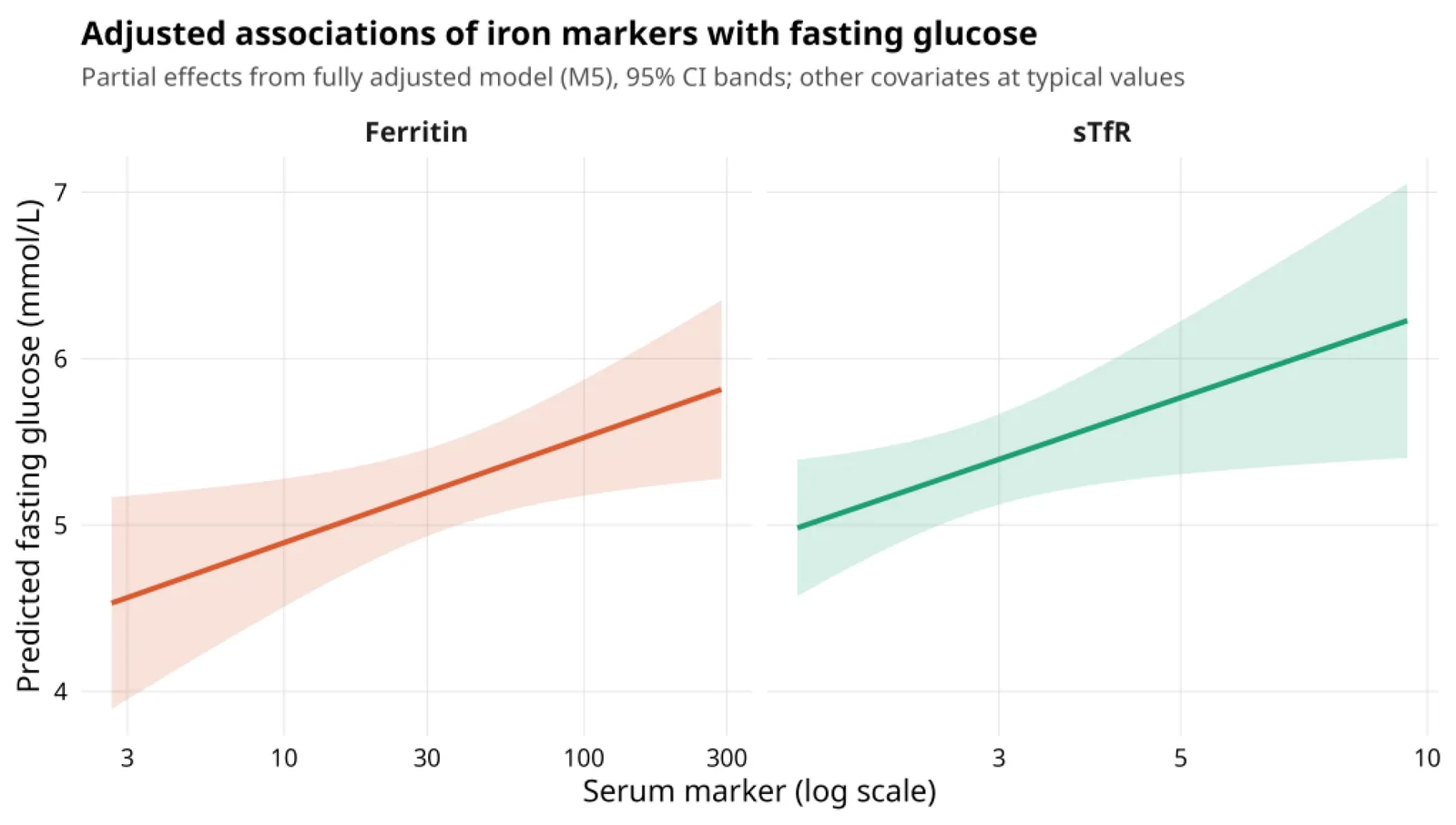
Adjusted associations of ferritin and sTfR with fasting glucose: partial effects from the fully adjusted model (M5) with 95% CI bands.

For log-transformed ferritin, the regression coefficient was β = 0.33 (95% CI 0.09–0.57; *p* = 0.007) in Model 2 and remained significant in the fully adjusted Model 5 (β = 0.27, 95% CI 0.04–0.50; *p* = 0.019) (Table 2). Similarly, log-transformed sTfR was independently associated with fasting glucose in Model 2 (β = 0.75, 95% CI 0.20–1.29; *p* = 0.008) and in the fully adjusted model (β = 0.73, 95% CI 0.20–1.25; *p* = 0.007) (Figure 2; stability across sequential models is shown in Figure S1).

For clinical interpretation, because the exposure variables were log-transformed, the fully adjusted estimates indicate that a two-fold increase in ferritin concentration corresponded to an average increase of approximately 0.19 mmol/L in fasting glucose, whereas a two-fold increase in sTfR corresponded to an average increase of approximately 0.51 mmol/L.

Importantly, the ferritin–glucose association was not explained by systemic inflammation. Inclusion of hs-CRP (transition from M2 to M3) did not attenuate the ferritin coefficient, arguing against elevated ferritin simply reflecting its role as an acute-phase protein in MetS. Likewise, the sTfR coefficient remained remarkably stable across Models M2–M5.

### 3.4. Metabolic Syndrome as a Binary Outcome

**Figure 3:**
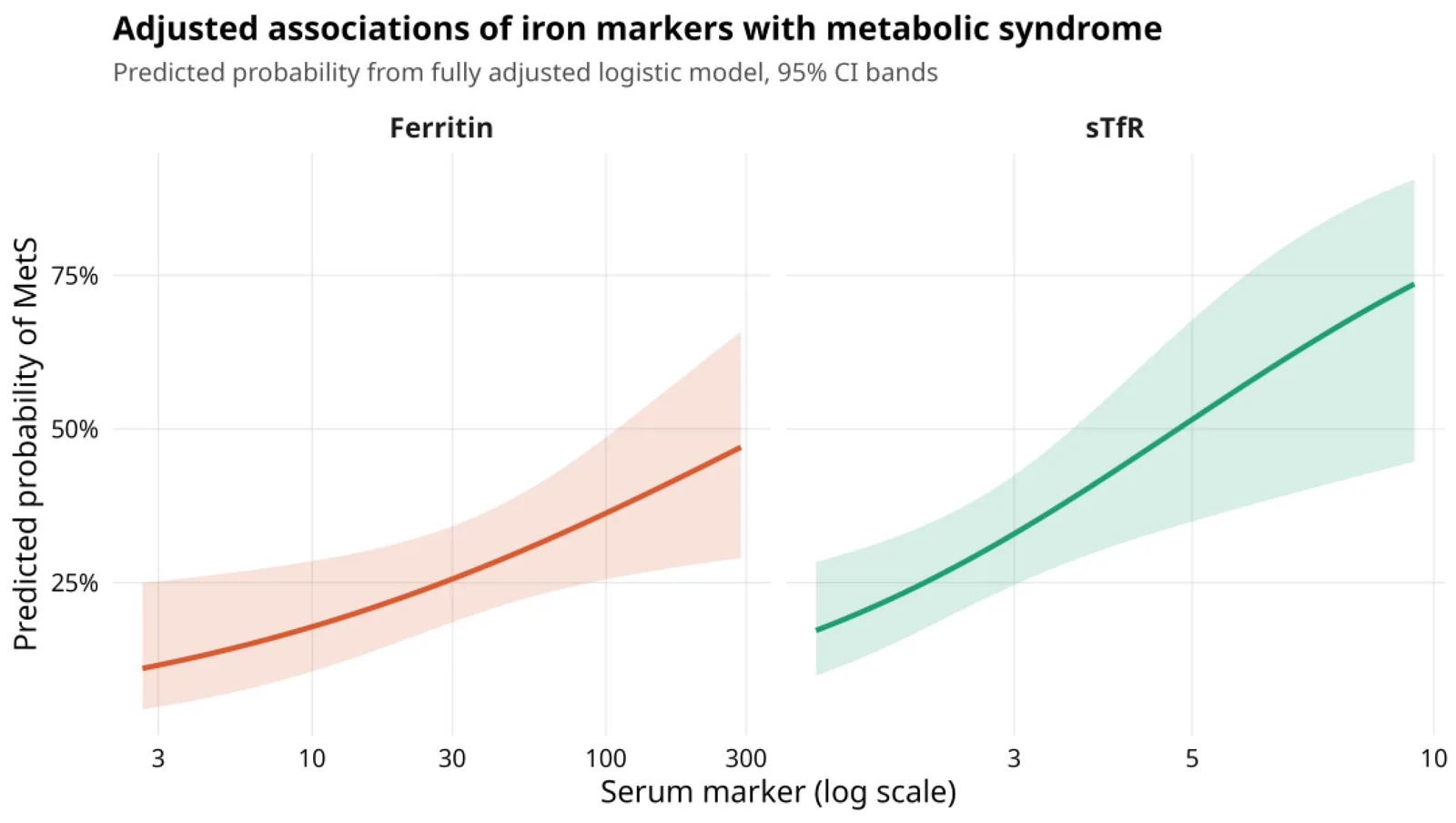
Adjusted associations of ferritin and sTfR with the probability of metabolic syndrome: predicted probability from the fully adjusted logistic model with 95% CI bands.

In multivariable logistic regression, both log-transformed ferritin and log-transformed sTfR were independently associated with the presence of MetS. Across sequential models, ferritin was associated with odds ratios ranging from 1.52 to 1.57 (*p* < 0.02), whereas sTfR showed substantially stronger associations (OR 4.1–4.5; *p* < 0.004) (Figure 3).

### 3.5. Suppression Effect of sTfR

Although sTfR showed no significant difference between MetS and non-MetS groups in the unadjusted comparison, it became strongly associated with MetS after multivariable adjustment (OR ≈ 4). This apparent paradox is explained by the negative correlation between ferritin and sTfR (Spearman ρ = −0.475, *p* < 0.001), reflecting their complementary physiological roles within iron metabolism. The suppression effect arises because ferritin and sTfR are inversely correlated (Spearman ρ = −0.475, *p* < 0.001) and capture opposing poles of iron homeostasis: ferritin reflects iron stores and rises with iron excess and inflammation, while sTfR reflects functional iron demand and rises when tissue iron availability is restricted. When entered jointly, each marker removes the shared variance attributable to general iron status, allowing the unique biological signal of the other to emerge. Specifically, controlling for ferritin removes the iron-replete background, revealing that elevated cellular iron demand (high sTfR) carries additional, ferritin-independent metabolic risk. Indeed, when ferritin was omitted from the model, the association between sTfR and MetS was not statistically significant (OR = 1.93, 95% CI 0.94–3.95, *p* = 0.072). After simultaneous adjustment for ferritin, the association strengthened markedly (OR = 4.12; *p* = 0.003), despite low variance inflation factors (VIFs 2.1 and 1.7), indicating no meaningful multicollinearity. Across nested models M2–M5, the sTfR odds ratio remained directionally consistent and statistically significant (OR range: 4.12–4.53, all *p* ≤ 0.004), arguing against estimate instability. Bootstrap confidence intervals for the sTfR odds ratio in Model M5 (2000 iterations, percentile method) were 95% CI: 1.73–14.28, confirming that the estimate does not cross the null. The direction of the sTfR–MetS association was therefore consistently positive throughout all model specifications; the suppression effect refers to the strengthening of this positive association after joint adjustment for ferritin, not a reversal of direction. From a clinical perspective, these findings indicate that sTfR is difficult to interpret in isolation and becomes informative only when evaluated together with ferritin.

The fully adjusted logistic model demonstrated good discrimination (AUC = 0.82, 95% CI 0.77–0.87) and satisfactory calibration (Hosmer–Lemeshow *p* = 0.085; Brier score = 0.170). However, calibration was assessed internally using the same dataset and therefore requires external validation. Adding iron biomarkers significantly improved model fit (likelihood ratio test *p* = 0.004), although the improvement in discrimination was modest (AUC 0.775 vs. 0.797; DeLong *p* = 0.055). Thus, iron biomarkers contribute additional prognostic information but only modestly improve patient risk classification.

Sex-Stratified Analyses. Formal sex-interaction terms (ferritin × sex and sTfR × sex) were added to both the linear and logistic regression models. Neither interaction reached statistical significance (ferritin × sex: glucose β = 0.043, *p* = 0.898; MetS OR = 0.58, *p* = 0.188). The sTfR × sex interaction could not be reliably estimated in the joint model due to quasi-complete separation in the male subgroup (*n* = 77). In sex-stratified models, the sTfR association with MetS was statistically significant among women (OR = 5.93, 95% CI 1.58–22.33, *p* = 0.008) and directionally consistent but non-significant among men (OR = 4.74, 95% CI 0.23–98.37, *p* = 0.315). Full results are presented in Supplementary Table S2.

### 3.6. Sensitivity Analyses

Non-glycaemic MetS outcome. In the fully adjusted Model M5, log-ferritin (OR = 1.33, 95% CI 0.95–1.85, *p* = 0.097) and log-sTfR (OR = 2.69, 95% CI 0.98–7.40, *p* = 0.055) showed attenuated associations with the non-glycaemic MetS composite (≥2 of 4 non-glucose criteria; *n* = 281, prevalence 59.1%), indicating partial glycaemic overlap (Supplementary Table S3).

Menopausal proxy. Women (*n* = 206) were stratified by age < 50 years (*n* = 110) and ≥50 years (*n* = 96). Among younger women, log-ferritin (OR = 2.49, 95% CI 1.30–4.75, *p* = 0.006) and log-sTfR (OR = 6.98, 95% CI 1.20–40.75, *p* = 0.031) remained independently associated with MetS. Among older women, point estimates were directionally consistent but non-significant (ferritin OR = 1.81, *p* = 0.119; sTfR OR = 8.94, *p* = 0.110), likely reflecting limited power (*n* = 96; Supplementary Table S4).

Liver disease covariate. After addition of a liver_disease covariate to Model M5, log-ferritin (OR = 1.52, 95% CI 1.08–2.14, *p* = 0.016) and log-sTfR (OR = 4.46, 95% CI 1.59–12.52, *p* = 0.004) remained virtually unchanged; liver_disease was not significantly associated with MetS (OR = 1.19, 95% CI 0.64–2.21, *p* = 0.581).

### 3.7. Secondary Outcomes

Among secondary continuous outcomes, only one association remained statistically significant after false discovery rate (FDR) correction: log-transformed ferritin was positively associated with log-transformed triglycerides in the fully adjusted model (Model 5; *p* = 0.0002; FDR = 0.001). No significant associations were observed with HDL cholesterol, waist circumference, systolic blood pressure, or diastolic blood pressure. These findings suggest that disturbances in iron metabolism are selectively associated with dysglycaemia and hypertriglyceridaemia rather than with all MetS components.

### 3.8. Path Analysis: Associative Mediation Through the Ferritin–GGT–Glucose Axis

**Figure 4:**
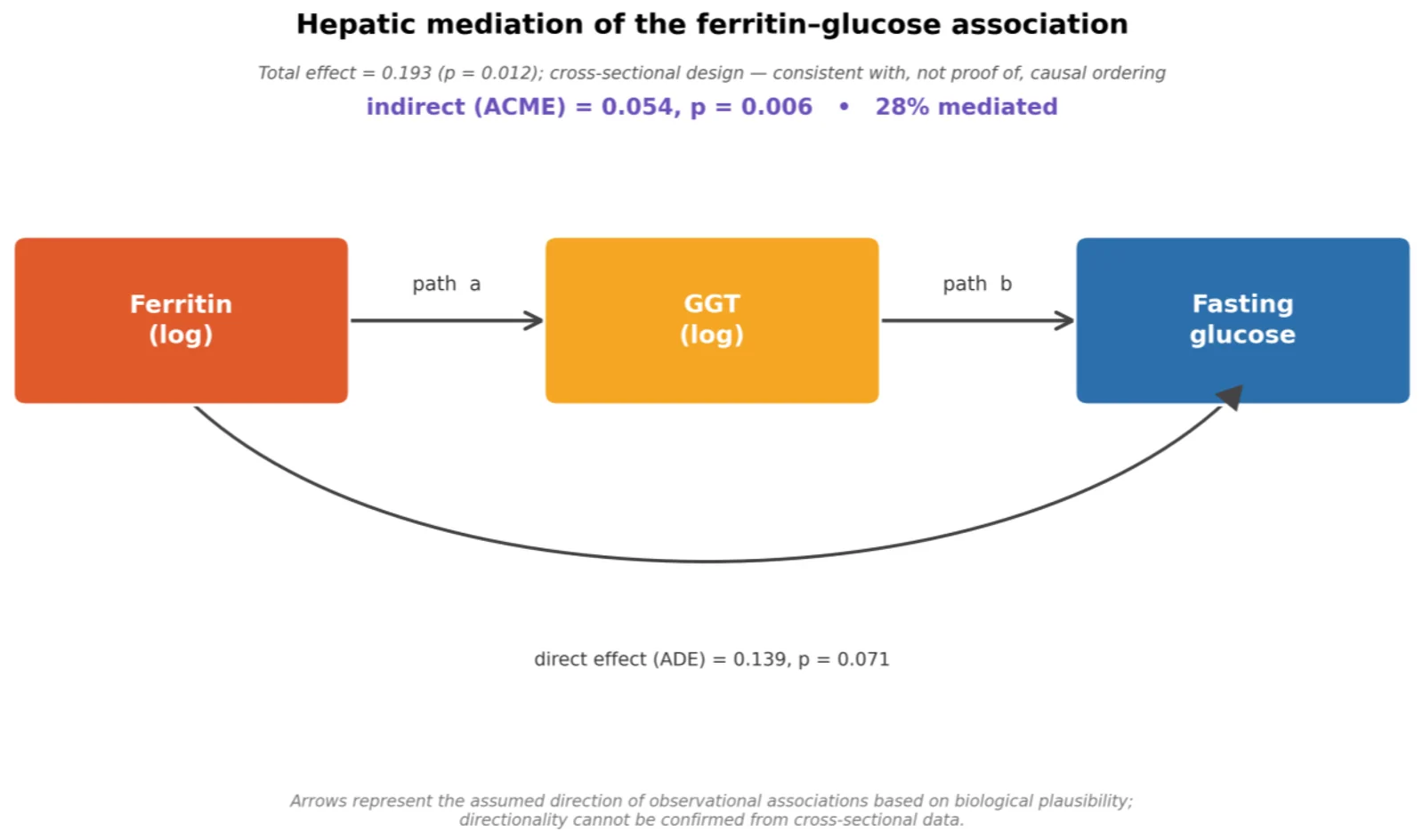
Path decomposition of the ferritin–glucose association: direct and indirect (via GGT) associative paths (bootstrap, 5000 resamples). Given the cross-sectional design, estimates are associative, not causal.

Path analysis (Figure 4) decomposed the ferritin–glucose association into direct and indirect (GGT-mediated) components. The indirect pathway through GGT was statistically significant (indirect association coefficient = 0.054, 95% bootstrap CI 0.011–0.118) and accounted for approximately 28% of the total ferritin–glucose association (total effect = 0.193, 95% CI 0.039–0.360). No comparable indirect pathway was identified for sTfR, suggesting that its association with fasting glucose is predominantly direct.

Because the exposure, proposed mediator, and outcome were measured simultaneously, these estimates represent associative rather than causal mediation. The assumed pathway direction was based on biological plausibility and cannot be statistically confirmed within a cross-sectional design. Therefore, these estimates should not be interpreted as evidence of causal mediation; prospective data are required to confirm directionality.

### 3.9. Diagnostic Performance

**Figure 5:**
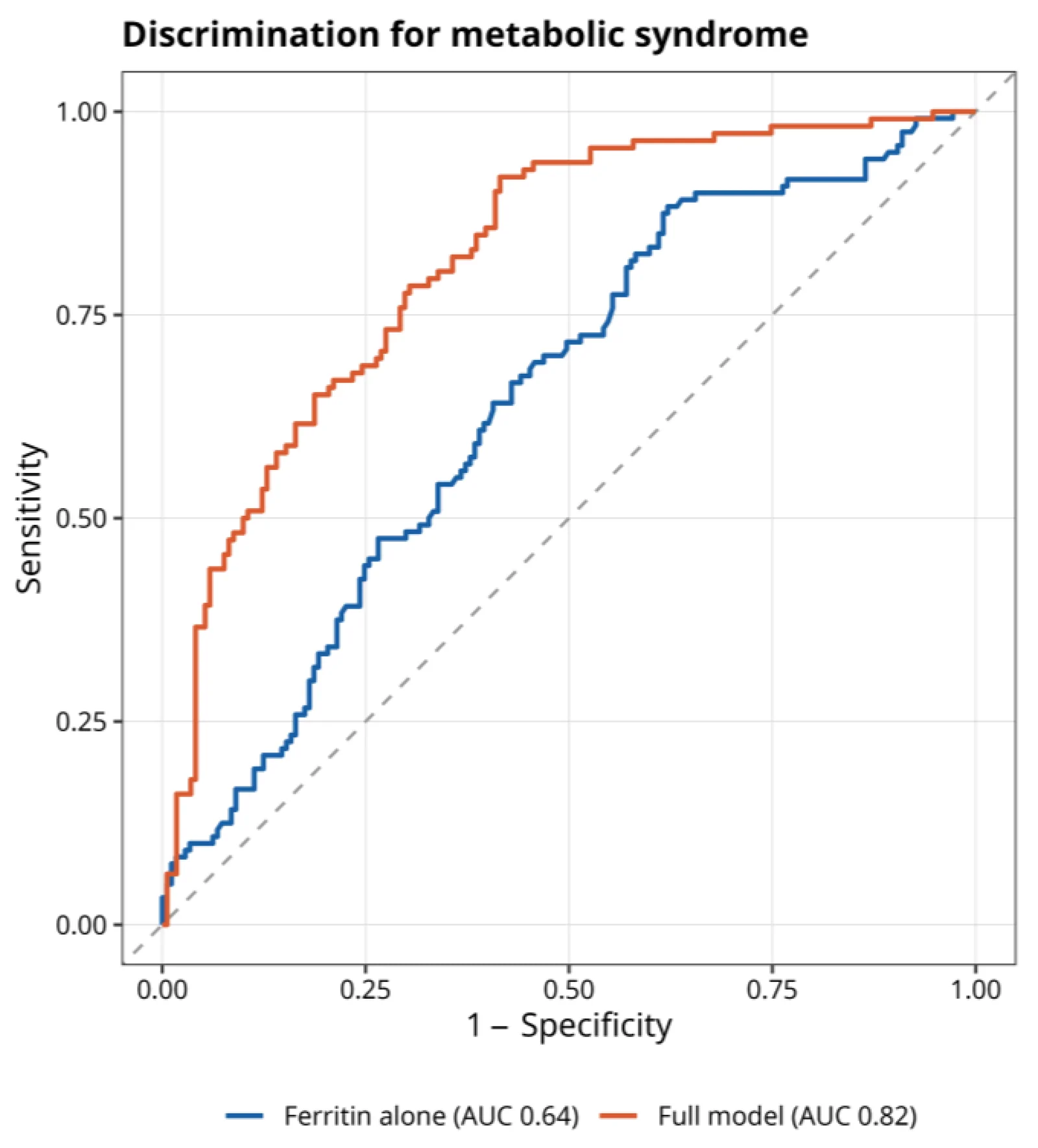
ROC curves for discrimination of metabolic syndrome: full model (AUC 0.82) versus ferritin alone (AUC 0.64).

The fully adjusted Model M5 demonstrated good discriminatory performance (AUC = 0.818, 95% CI 0.767–0.866). Ferritin alone showed limited discrimination (AUC = 0.633, 95% CI 0.569–0.695). Sex-stratified analysis showed modest discrimination in women (AUC = 0.652, 95% CI 0.571–0.732) and no discrimination in men (AUC = 0.500, 95% CI 0.370–0.630). Among men (*n* = 77), MetS prevalence was 49.4% (*n* = 38 events), and median ferritin was virtually identical in those with and without MetS (97.6 vs. 93.9 µg/L, *p* = 0.998), indicating the complete absence of a metabolic ferritin signal in this subgroup. Ferritin AUC in men was 0.500 (95% CI 0.370–0.630), confirming no discriminatory ability. The absence of discrimination is therefore not attributable to an insufficient number of MetS events but rather to the lack of a ferritin difference between metabolic groups. The full model significantly outperformed ferritin alone (DeLong test: *p* < 0.001). All AUC values were estimated with bootstrap confidence intervals (2000 iterations; Figure 5).

### 3.10. Subclinical Gradient of Biomarkers (Exploratory Analysis)

**Figure 6:**
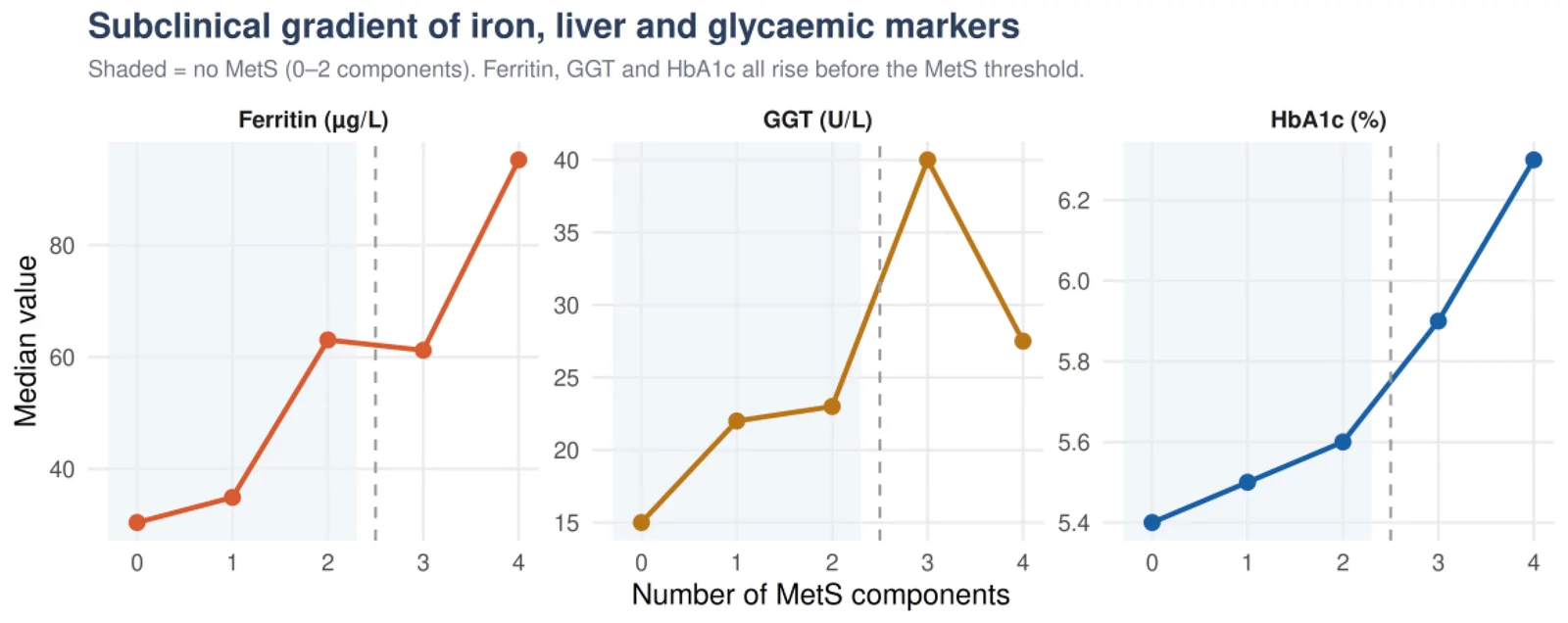
Subclinical gradient of markers. Median ferritin, GGT, and HbA1c by number of MetS components; shaded area = no MetS (0–2 components), dashed line = MetS threshold (≥3 components). All three markers rise within the subclinical range. sTfR is not shown (no significant trend). Non-monotonicity of GGT at 3–4 components reflects small subgroup sizes.

Exploratory analyses demonstrated progressive increases in ferritin, GGT, and HbA1c with an increasing number of MetS components (Figure 6). Across the entire cohort, Spearman trend tests were significant for ferritin (ρ = 0.26), GGT (ρ = 0.36), and HbA1c (ρ = 0.47) (all *p* < 0.001), whereas no significant trend was observed for sTfR (ρ = 0.11; *p* = 0.070).

Importantly, these trends persisted among participants without MetS (0–2 components). Ferritin (ρ = 0.19; *p* = 0.003), GGT (ρ = 0.28; *p* < 0.001), and HbA1c (ρ = 0.24; *p* < 0.001) increased significantly even within the subclinical range, whereas sTfR again showed no significant trend (ρ = 0.08; *p* = 0.198). These findings are consistent with a hypothesis that ferritin, GGT, and HbA1c covary with accumulating metabolic burden within the subclinical range; however, the cross-sectional design precludes any inference about temporal sequence, and reverse causality cannot be excluded.

## 4. Discussion

This cross-sectional study of adults from Southern Kazakhstan yielded four principal findings. First, both axes of iron metabolism—the iron storage marker ferritin and the functional marker soluble transferrin receptor (sTfR)—were independently associated with fasting glucose and metabolic syndrome (MetS). These associations remained robust after adjustment for systemic inflammation, dietary inflammatory potential, and the *HFE* H63D genotype. Second, the association between sTfR and MetS emerged only after simultaneous adjustment for ferritin, revealing a suppression effect of ferritin. Third, path-analytic decomposition indicated that approximately 28% of the ferritin–glucose association was accounted for by an observational indirect path through GGT, whereas no analogous pathway was observed for sTfR. Finally, ferritin, GGT, and HbA1c increased progressively with the number of MetS components, even among individuals without MetS, suggesting that these markers covary with metabolic burden before the diagnostic threshold for MetS is reached; temporal ordering cannot be established from cross-sectional data.

### 4.1. Ferritin Is Associated with Fasting Glucose and Metabolic Syndrome

Ferritin was positively associated with both fasting glucose (β = 0.27, 95% CI 0.04–0.50 in the fully adjusted model) and MetS (OR = 1.52, 95% CI 1.08–2.14). The magnitude of these associations is consistent with previous reports. In the KORA F4 study, individuals in the highest ferritin quartile had higher odds of impaired glucose tolerance (OR = 2.08) and T2DM (OR = 1.98) [17]. Likewise, several meta-analyses have consistently demonstrated positive associations of ferritin with both MetS [18] and T2DM [2,19].

Importantly, the observed associations remained significant after adjustment for hs-CRP, E-DII, and the *HFE* H63D genotype (Models M3–M4). These findings suggest that elevated ferritin is not merely a consequence of systemic inflammation but provides information on metabolic risk that is independent of inflammatory status and common genetic variation in iron metabolism.

### 4.2. Association Between sTfR and Glycaemia

The functional iron marker sTfR was independently associated with fasting glucose (β = 0.73, 95% CI 0.20–1.26) and MetS (OR = 4.53, 95% CI 1.64–12.52), even after accounting for ferritin. Previous studies have reported inconsistent findings regarding sTfR. Some observed positive associations with insulin resistance but not with MetS [4]; the EPIC-Potsdam study found no significant association with incident T2DM [5], whereas another study reported that the association with T2DM was present only among individuals with obesity [18].

Our results provide a possible explanation for these discrepancies. Ferritin and sTfR change in opposite physiological directions: sTfR increases in iron deficiency, whereas ferritin increases with iron overload and metabolic dysfunction. Consequently, when sTfR is analysed alone, these opposing influences partially cancel each other. In our cohort, median sTfR decreased across ferritin tertiles from 3.23 to 2.37 mg/L, while the prevalence of MetS increased from 23% to 52%.

This suppression effect became evident in multivariable modelling. Without ferritin in the model, sTfR was not significantly associated with MetS (OR = 1.93, 95% CI 0.94–3.95). After adjustment for ferritin, however, the association strengthened markedly (OR = 4.12, 95% CI 1.62–10.48). This finding cannot be explained by multicollinearity, as variance inflation factors remained low (VIF = 2.1 and 1.7). Similar observations were reported in the EPIC-InterAct study, where ferritin and transferrin were strongly inversely correlated, yet both independently predicted incident T2DM [20].

### 4.3. Subclinical Gradient of Iron, Liver, and Glycaemic Biomarkers

Ferritin, GGT, and HbA1c increased monotonically with the number of MetS components (ρ = 0.26, 0.36, and 0.47, respectively; all *p* < 0.001). Importantly, these trends persisted among individuals without MetS (0–2 components): ferritin (ρ = 0.19, *p* = 0.003), GGT (ρ = 0.28, *p* < 0.001), and HbA1c (ρ = 0.24, *p* < 0.001) all increased significantly before the diagnostic threshold for MetS was reached.

These findings are consistent with the hypothesis that iron–liver–glycaemia marker co-elevation accompanies early metabolic deterioration, although reverse causality and residual confounding cannot be excluded in a cross-sectional design. This observation has important clinical implications because individuals with two MetS components are currently classified as not having MetS despite carrying substantially elevated metabolic risk, which increases progressively with each additional component [21]. Furthermore, interventions initiated before overt disease develops have been shown to reduce the incidence of T2DM by more than 50% [22]. Biomarkers that change before diagnostic thresholds are reached may therefore facilitate earlier identification of high-risk individuals.

### 4.4. GGT Mediates Part of the Ferritin–Glucose Association

When GGT was added to the fully adjusted regression model (transition from M4 to M5), the ferritin coefficient decreased by approximately 18% (from β = 0.33 to β = 0.27), whereas the sTfR coefficient remained essentially unchanged. Path analysis confirmed a significant indirect pathway through GGT (indirect effect = 0.054, 95% bootstrap CI 0.011–0.115), accounting for approximately 28% of the total ferritin–glucose association. This result was independent of whether sTfR was included in the model. No comparable indirect pathway was identified for sTfR.

Biologically, these findings are plausible because the liver is both the principal iron storage organ and a key site of insulin resistance, while GGT is a well-established predictor of both MetS [11] and T2DM [12]. Additional support comes from the secondary outcome analyses. After false discovery rate correction, the only significant association that remained was between ferritin and triglycerides (FDR = 0.001), whereas no significant associations were observed with the remaining MetS components. Notably, both fasting glucose and triglycerides are strongly influenced by hepatic metabolism.

Furthermore, analyses of the extended iron panel demonstrated that higher glycaemia was associated with increased iron storage but lower circulating iron availability. Specifically, TSAT and serum iron were inversely associated with fasting glucose (β = −0.041 and −0.063, respectively; both FDR = 0.028), whereas ferritin showed a positive association. A similar inverse association between TSAT and glycaemia was previously reported in the KORA F4 study [18].

### 4.5. Diagnostic Value of Iron Metabolism Biomarkers

From a clinical perspective, our findings indicate that individual iron metabolism biomarkers should not be interpreted in isolation. Ferritin alone demonstrated limited ability to discriminate participants with MetS (AUC = 0.64), particularly among men (AUC = 0.50), whereas discrimination was only modest among women (AUC = 0.65).

The multivariable model incorporating age, sex, BMI, ferritin, sTfR, and the remaining study variables demonstrated good discrimination (AUC = 0.82, 95% CI 0.77–0.87) and satisfactory calibration (Hosmer–Lemeshow *p* = 0.085; Brier score = 0.170).

A key practical implication is that both axes of iron metabolism should be evaluated simultaneously. Ferritin and sTfR together discriminated MetS better than ferritin alone (AUC 0.689 vs. 0.633), with further improvement after adding GGT (AUC = 0.701). In contrast, combining both biomarkers into a single calculated index reduced diagnostic performance: the Cook iron stores index yielded an AUC of only 0.606 (*p* < 0.001 compared with ferritin alone). Thus, the two biomarkers should be interpreted jointly rather than collapsed into a single composite measure.

### 4.6. Comparison with Asian Studies

Our findings are consistent with those reported in other Asian populations. In a cross-sectional study conducted among adults in eight Chinese cities, serum ferritin was independently associated with metabolic syndrome (MetS), regardless of place of residence or dietary patterns [23]. A systematic review and dose–response meta-analysis of several Asian cohorts also demonstrated an association between serum ferritin and MetS [24]. Similarly, using data from the China National Nutrition and Health Survey, Ren et al. found that ferritin was strongly associated with metabolically unhealthy phenotypes, whereas sTfR alone showed no significant association [25]. This lack of an independent effect of sTfR is consistent with our observation that sTfR becomes statistically significant only after mutual adjustment for ferritin, supporting the concept that sTfR is not independently informative without considering iron stores. The magnitude of the association between ferritin and MetS in the present study was comparable to estimates reported in East Asian cohorts.

### 4.7. Strengths and Limitations

The principal strength of this study is that it represents one of the first comprehensive investigations of iron metabolism biomarkers and MetS in a Central Asian (Kazakh) population [15,17]. Because findings from European and East Asian cohorts may not be directly generalisable to this region, our study addresses an important gap in the literature.

Several limitations should be acknowledged.

First, the study design is cross-sectional. Causal inference is therefore not possible. All associations, including the path analysis, are observational. Reverse causality cannot be excluded. Second, over-adjustment in the joint ferritin–sTfR model cannot be ruled out. VIF values were low (2.09 and 1.72), and bootstrap resampling supported estimate stability (95% CI: 1.73–14.28). However, prospective data are needed to confirm the independence of the sTfR signal. Third, insulin and HOMA-IR were not measured. The mechanistic link between iron excess and impaired insulin signalling therefore remains speculative.

Fourth, the E-DII was not validated in the Kazakh population. Dietary variability in this sample was limited (median E-DII: 0.065 vs. 0.137 in non-MetS and MetS groups). This reduces the statistical contribution of E-DII as a covariate but does not indicate a calibration failure. Fifth, the extended iron panel was available in only 207 of 297 participants. Selection was random, but baseline differences between subgroups were observed. These analyses should be treated as exploratory. Sixth, participants with liver or gallbladder disease were not formally excluded. A sensitivity analysis with a liver_disease covariate showed no material change in results, suggesting hepatobiliary confounding was not substantial. Seventh, subgroup analyses were conducted in small subsamples. Findings should be interpreted as hypothesis-generating, not confirmatory. Finally, the model was internally validated. External validation in an independent Central Asian cohort has not been performed. The reported AUC may overestimate true performance in new samples.

## 5. Conclusions

In this Central Asian cohort, both iron storage (ferritin) and functional iron status (sTfR) were independently associated with metabolic syndrome and glycaemia beyond the effects of systemic inflammation. The association between ferritin and fasting glucose showed an observational link to GGT, which accounted for approximately 28% of the ferritin–glucose path-analytic estimate, whereas ferritin, GGT, and HbA1c increased progressively across the continuum of metabolic risk even among individuals below the diagnostic threshold for MetS. These findings support the combined assessment of iron metabolism and liver function biomarkers for metabolic risk stratification and warrant confirmation in prospective longitudinal studies.

## Figures and Tables

**Table 1:** Participant characteristics by metabolic syndrome status.

Characteristic	Overall ^1^ *(n* = 297)	No MetS ^1^ (*n* = 177)	MetS ^1^ (*n* = 120)	*p*-Value ^2^
Sex: female, *n* (%)	206 (72.8)	132 (77.2)	74 (66.1)	0.042
Age, years	48.0 (37.0, 57.0)	44.0 (35.0, 54.0)	52.5 (43.5, 60.0)	<0.001
BMI, kg/m^2^	28.7 (25.2, 32.0)	27.3 (24.2, 30.5)	31.2 (28.2, 33.8)	<0.001
Waist circumference, cm	98.0 (89.0, 106.0)	92.0 (84.0, 100.0)	104.0 (98.0, 111.0)	<0.001
SBP, mmHg	119.0 (100.0, 129.0)	110.0 (100.0, 120.0)	122.0 (110.0, 132.8)	<0.001
DBP, mmHg	74.0 (70.0, 80.0)	70.0 (70.0, 80.0)	80.0 (70.0, 90.0)	<0.001
Fasting glucose, mmol/L	4.9 (4.6, 5.4)	4.7 (4.5, 5.0)	5.3 (4.8, 6.2)	<0.001
HbA1c, %	5.5 (5.2, 5.8)	5.4 (5.1, 5.6)	5.7 (5.4, 6.2)	<0.001
HDL-C, mmol/L	1.2 (1.0, 1.5)	1.4 (1.1, 1.6)	1.1 (0.9, 1.2)	<0.001
Triglycerides, mmol/L	1.2 (0.8, 1.6)	0.9 (0.7, 1.2)	1.7 (1.2, 2.1)	<0.001
Ferritin, µg/L	49.9 (15.7, 111.1)	31.6 (12.0, 82.9)	65.9 (27.4, 126.5)	<0.001
Serum iron, µmol/L	14.4 (11.0, 18.1)	14.4 (10.6, 17.9)	14.7 (11.3, 18.4)	0.373
Transferrin, g/L	2.7 (2.5, 3.1)	2.7 (2.5, 3.1)	2.7 (2.5, 3.0)	0.563
TSAT, %	21.0 (15.0, 28.0)	20.0 (14.0, 28.0)	21.0 (16.0, 27.0)	0.325
sTfR, mg/L	2.7 (2.2, 3.3)	2.6 (2.2, 3.3)	2.8 (2.3, 3.3)	0.145
hs-CRP, mg/L	1.7 (0.8, 3.1)	1.4 (0.6, 2.8)	2.2 (1.0, 3.9)	0.002
GGT, U/L	21.0 (14.0, 33.5)	18.0 (13.5, 28.0)	27.0 (19.0, 43.5)	<0.001
ALT, U/L	19.8 (15.3, 30.2)	18.0 (14.2, 26.7)	23.1 (17.6, 36.9)	<0.001
E-DII score	0.10 (−0.05, 0.23)	0.07 (−0.06, 0.20)	0.14 (0.01, 0.25)	0.002
HFE H63D carrier, *n* (%)	55 (19.4)	31 (18.1)	24 (21.4)	0.540

1. Continuous variables, median (Q1, Q3); categorical, *n* (%). 2. Wilcoxon and Fisher exact tests; BMI, body mass index; SBP/DBP, systolic/diastolic blood pressure; HDL-C, high-density lipoprotein cholesterol; TSAT, transferrin saturation; sTfR, soluble transferrin receptor; hs-CRP, high-sensitivity C-reactive protein; GGT, gamma-glutamyl transferase; ALT, alanine aminotransferase; E-DII, energy-adjusted dietary inflammatory index; HbA1c, glycated haemoglobin.

**Table 2:** Ferritin and sTfR coefficients across nested multivariable models: linear regression on fasting glucose (β, HC3-robust) and logistic regression on metabolic syndrome (OR, HC3-robust 95% CI).

Model	Covariates Added	Fer β	*p*	sTfR β	*p*	Fer OR	*p*	sTfR OR	*p*
M1	+Ferritin	0.191	0.025	—	—	1.20	0.144	—	—
M2	+sTfR	0.331	0.007	0.747	0.008	1.56	0.003	4.12	0.003
M3	+hs-CRP	0.347	0.004	0.781	0.006	1.57	0.004	4.22	0.003
M4	+E-DII, H63D	0.334	0.005	0.763	0.005	1.54	0.010	4.53	0.004
M5	+GGT	0.273	0.020	0.729	0.007	1.52	0.015	4.53	0.004

## Data Availability

De-identified participant data supporting the findings of this study are available from the corresponding author upon reasonable request, subject to institutional ethics approval and applicable national data protection regulations.

## Supplementary Materials

The following supporting information can be downloaded at https://www.mdpi.com/article/10.3390/diagnostics16172773/s1. Figure S1: Robustness of iron-marker associations across nested models. Table S1. Comparison of baseline characteristics between participants with and without extended iron panel data. Table S2. Sex-stratified associations of log-ferritin and log-sTfR with metabolic syndrome, and formal sex-interaction tests. Table S3. Sensitivity analysis: associations of log-ferritin and log-sTfR with the primary MetS outcome and a non-glycaemic MetS composite. Table S4. Sensitivity analysis: associations of log-ferritin and log-sTfR with metabolic syndrome, stratified by age group as a proxy for menopausal status.
