# Supplementary material for: Ferritin and Soluble Transferrin Receptor Beyond Inflammation: Independent Associations with Fasting Glucose and Metabolic Syndrome in a Central Asian Cohort
Source: Diagnostics (Basel). 2026 Aug 28;16(17):2773. doi: 10.3390/diagnostics16172773 (PMC13565190; doi:10.3390/diagnostics16172773)
Supplement: Supplementary file 1 [file diagnostics-16-02773-s001.zip › diagnostics-4508967-supplementary.pdf]

Supplementary Materials

Storage and functional iron markers and metabolic syndrome in a Central Asian cohort

Figure S1

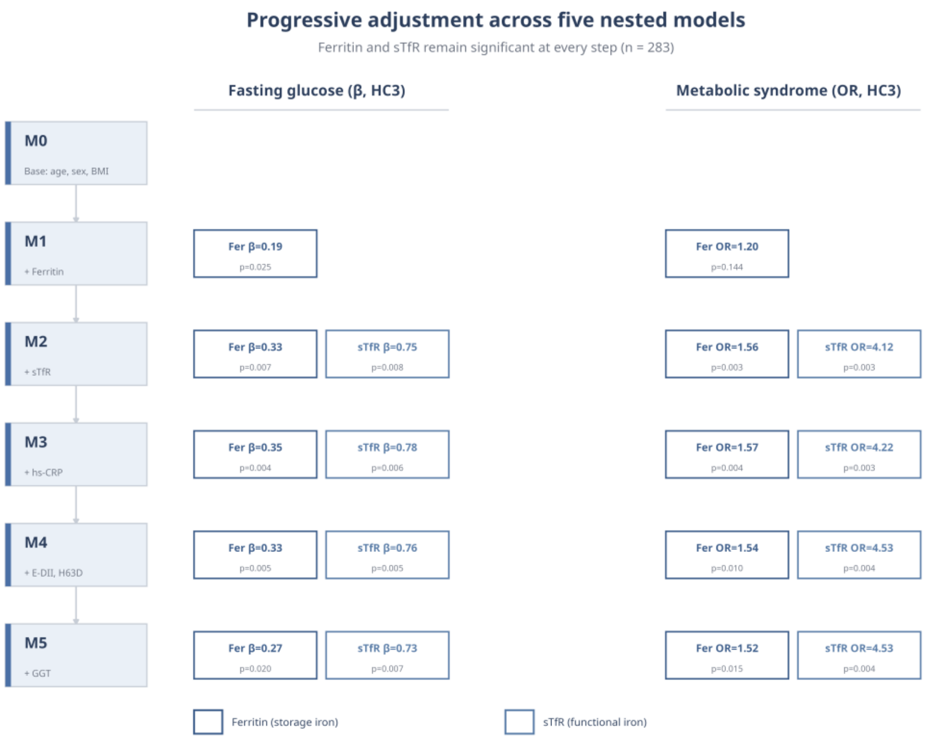

Figure S1. *Robustness of iron-marker associations across nested models. Coefficients for log-transformed ferritin (storage iron) and log-transformed sTfR (functional iron) with fasting glucose (β, left) and metabolic syndrome (OR, right) across progressively adjusted models M0–M5 (HC3 robust estimates, n = 283). Both markers remain significant at every step. Model M1 includes ferritin only; sTfR enters from M2, which explains the weaker ferritin estimate in M1 (for MetS, OR = 1.20, p = 0.144). Adjustment blocks: M0 = age, sex, BMI; M1 = + ferritin; M2 = + sTfR; M3 = + hs-CRP; M4 = + E-DII and HFE H63D; M5 = + GGT.*

Supplementary Table S1. *Comparison of baseline characteristics between participants with and without extended iron panel data (serum iron, transferrin, TSAT).*

Characteristic	With panel (n=207)	Without panel (n=90)	p
Age, years (median)	51	40	<0.001
BMI, kg/m² (median)	29.3	27.2	0.004
Fasting glucose, mmol/L (median)	4.94	4.78	<0.001
Ferritin, µg/L (median)	53.5	29.9	0.018
MetS prevalence, %	45.4	28.9	—

Supplementary Table S2. *Sex-stratified associations of log-ferritin and log-sTfR with metabolic syndrome, and formal sex-interaction tests.*

Analysis	Marker	Estimate	95% CI	p
Interaction: glucose	ferritin × sex	β = 0.043	—	0.898
Interaction: MetS	ferritin × sex	OR = 0.58	—	0.188
Interaction: MetS	sTfR × sex	quasi-separation	—	—
Stratified: Women (n=206)	log-sTfR	OR = 5.93	1.58–22.33	0.008
Stratified: Men (n=77)	log-sTfR	OR = 4.74	0.23–98.37	0.315

Supplementary Table S3. *Sensitivity analysis: associations of log-ferritin and log-sTfR with the primary MetS outcome and a non-glycaemic MetS composite (≥2 of 4 non-glucose criteria; elevated waist circumference, hypertriglyceridaemia, low HDL-C, and elevated blood pressure). Fully adjusted Model M5 (n = 283 and n = 281, respectively).*

Outcome	Ferritin OR [95% CI]	p	sTfR OR [95% CI]	p
Primary MetS (≥3 of 5 criteria)	1.52 [1.08–2.14]	0.015	4.53 [1.64–12.52]	0.004
Non-glycaemic MetS (≥2 of 4 criteria)	1.33 [0.95–1.85]	0.097	2.69 [0.98–7.40]	0.055

Supplementary Table S4. *Sensitivity analysis: associations of log-ferritin and log-sTfR with metabolic syndrome, stratified by age group as a proxy for menopausal status (women only, n = 206). Fully adjusted Model M5 (excluding age as a stratification variable).*

Age group	n	Ferritin OR [95% CI]	p	sTfR OR [95% CI]	p
<50 years (pre-menopausal proxy)	110	2.49 [1.30–4.75]	0.006	6.98 [1.20–40.75]	0.031
≥50 years (post-menopausal proxy)	96	1.81 [0.86–3.81]	0.119	8.94 [0.61–131.77]	0.110
